# Validation of the Klinrisk chronic kidney disease progression model in the FIDELITY population

**DOI:** 10.1093/ckj/sfae052

**Published:** 2024-03-06

**Authors:** Navdeep Tangri, Thomas Ferguson, Silvia J Leon, Stefan D Anker, Gerasimos Filippatos, Bertram Pitt, Peter Rossing, Luis M Ruilope, Alfredo E Farjat, Youssef M K Farag, Patrick Schloemer, Robert Lawatscheck, Katja Rohwedder, George L Bakris

**Affiliations:** Department of Internal Medicine, Max Rady College of Medicine, University of Manitoba, Winnipeg, Manitoba, Canada; Seven Oaks Hospital Chronic Disease Innovation Centre, Winnipeg, Manitoba, Canada; Department of Internal Medicine, Max Rady College of Medicine, University of Manitoba, Winnipeg, Manitoba, Canada; Seven Oaks Hospital Chronic Disease Innovation Centre, Winnipeg, Manitoba, Canada; Seven Oaks Hospital Chronic Disease Innovation Centre, Winnipeg, Manitoba, Canada; University of Manitoba, Community Health Sciences, Winnipeg, Manitoba, Canada; Department of Cardiology (CVK) of German Heart Center Charité; German Centre for Cardiovascular Research (DZHK) partner site Berlin, Charité Universitätsmedizin, Berlin, Germany; Institute of Heart Diseases, Wroclaw Medical University, Wroclaw, Poland; National and Kapodistrian University of Athens, School of Medicine, Department of Cardiology, Attikon University Hospital, Athens, Greece; Department of Medicine, University of Michigan School of Medicine, Ann Arbor, MI, USA; Steno Diabetes Center Copenhagen, Herlev, Denmark; Department of Clinical Medicine, University of Copenhagen, Copenhagen, Denmark; Cardiorenal Translational Laboratory and Hypertension Unit, Institute of Research imas12, Madrid, Spain; CIBER-CV, Hospital Universitario 12 de Octubre, Madrid, Spain; Faculty of Sport Sciences, European University of Madrid, Madrid, Spain; Research and Development, Clinical Data Sciences and Analytics, Bayer PLC, Reading, UK; US Medical Affairs, Bayer US LLC Pharmaceuticals, Whippany, NJ, USA; Statistics and Data Insights, Bayer AG, Berlin, Germany; Cardiology and Nephrology Clinical Development, Bayer AG, Berlin, Germany; Cardio-Renal Medical Affairs Department, Bayer AG, Berlin, Germany; Department of Medicine, University of Chicago Medicine, Chicago, IL, USA

**Keywords:** chronic kidney disease, external validation, kidney failure, Klinrisk, laboratory-based prediction model

## Abstract

**Background:**

Chronic kidney disease (CKD) affects >800 million individuals worldwide and is often underrecognized. Early detection, identification and treatment can delay disease progression. Klinrisk is a proprietary CKD progression risk prediction model based on common laboratory data to predict CKD progression. We aimed to externally validate the Klinrisk model for prediction of CKD progression in FIDELITY (a prespecified pooled analysis of two finerenone phase III trials in patients with CKD and type 2 diabetes). In addition, we sought to identify evidence of an interaction between treatment and risk.

**Methods:**

The validation cohort included all participants in FIDELITY up to 4 years. The primary and secondary composite outcomes included a ≥40% decrease in estimated glomerular filtration rate (eGFR) or kidney failure, and a ≥57% decrease in eGFR or kidney failure. Prediction discrimination was calculated using area under the receiver operating characteristic curve (AUC). Calibration plots were calculated by decile comparing observed with predicted risk.

**Results:**

At time horizons of 2 and 4 years, 993 and 1795 patients experienced a primary outcome event, respectively. The model predicted the primary outcome accurately with an AUC of 0.81 for 2 years and 0.86 for 4 years. Calibration was appropriate at both 2 and 4 years, with Brier scores of 0.067 and 0.115, respectively. No evidence of interaction between treatment and risk was identified for the primary composite outcome (*P* = .31).

**Conclusions:**

Our findings demonstrate the accuracy and utility of a laboratory-based prediction model for early identification of patients at the highest risk of CKD progression.

KEY LEARNING POINTS **What was known:**

- Chronic kidney disease (CKD) affects >800 million individuals worldwide and is frequently underrecognized.
- Early detection, diagnosis, and treatment can lead to improved outcomes and delays in disease progression.
- Validated risk prediction models can help identify patients who would receive the greatest net benefit from targeted treatment interventions and enhance patient care.

**This study adds:**

- The Klinrisk machine learning model accurately predicted CKD progression in individuals with CKD associated with type 2 diabetes.
- This analysis validates the Klinrisk model in a large, well-characterized, global, randomized clinical trial population across all stages of CKD, demonstrating good discrimination and calibration.

**Potential impact:**

- These findings support the potential clinical utility of the Klinrisk machine learning risk prediction model for early identification of patients at highest risk of CKD progression.
- Early identification of the risk of CKD progression coupled with initiation of appropriate treatment has the potential of preventing the life-limiting risk of kidney failure and other complications.

## INTRODUCTION

Chronic kidney disease (CKD) affects >800 million individuals worldwide [1] and is often underrecognized, with the prevalence of undiagnosed stage 3 CKD estimated to be 61.6–95.5% [2]. At advanced stages, when diagnosis is more common [3], the majority of kidney function is lost [4] and the window for disease-modifying therapy is narrow. Clinical practice guidelines recommend kidney function should be regularly monitored in patients with diabetes, and early detection and risk stratification are prioritized to ensure early diagnosis and treatment [4–6].

Klinrisk is a proprietary risk prediction model that uses a single time point measure of routinely collected laboratory data to predict CKD progression [7]. The model has been externally validated and found to be highly accurate for predictions evaluated up to 5 years for the composite outcome of a 40% decrease in estimated glomerular filtration rate (eGFR) or kidney failure [7]. Although the model is promising, it is important to externally validate it in an independently derived dataset.

FInerenone in chronic kiDney diseasE and type 2 diabetes: Combined FIDELIO-DKD and FIGARO-DKD Trial programme analYsis (FIDELITY) is a prespecified pooled analysis of the complementary phase III FInerenone in reducing kiDnEy faiLure and dIsease prOgression in Diabetic Kidney Disease (FIDELIO-DKD) and FInerenone in reducinG cArdiovascular moRtality and mOrbidity in Diabetic Kidney Disease (FIGARO-DKD) trials. These investigated the efficacy of the non-steroidal mineralocorticoid receptor antagonist finerenone on cardiovascular (CV) and kidney outcomes in patients with CKD and type 2 diabetes (T2D). To date, FIDELITY provides a dataset of one of the largest clinical trial populations of patients (*N* = 13 026) with early-to-late stages of CKD and T2D [8–10].

This analysis aimed to externally validate the Klinrisk model for the prediction of a primary composite kidney outcome of a ≥40% decrease in eGFR or kidney failure (defined as end-stage kidney disease or an eGFR <15 ml/min/1.73 m^2^) as well as a secondary composite kidney outcome of a ≥57% decrease in eGFR or kidney failure, up to 4 years post randomization in FIDELITY. Additionally, this analysis aimed to identify categories of risk that correspond with the greatest net benefit for targeted treatment intervention in patients with CKD and T2D.

## MATERIALS AND METHODS

### Study population

#### Validation cohort

The dataset included all participants in the FIDELITY prespecified pooled analysis, which combined individual patient-level data from the FIDELIO-DKD (NCT02540993) and FIGARO‑DKD (NCT02545049) phase III, multicentre, double-blind trials. Eligible participants were adults (≥18 years) with CKD [urine albumin:creatinine ratio (UACR) ≥3.4–<33.9 mg/mmol and eGFR ≥25–≤90 ml/min/1.73 m^2^ or a UACR ≥33.9–≤565.6 mg/mmol and eGFR ≥25 ml/min/1.73 m^2^] and T2D, receiving maximum tolerated doses of renin–angiotensin system therapy with a serum potassium level ≤4.8 mmol/l. Key exclusion criteria included diagnosis of symptomatic chronic heart failure with reduced ejection fraction (i.e. a class IA recommendation for mineralocorticoid receptor antagonist treatment) [8–10]. In the FIDELIO‑DKD and FIGARO-DKD studies, time to kidney failure (defined as end-stage kidney disease [initiation of long-term dialysis for ≥90 days or kidney transplant] or eGFR <15 ml/min/1.73 m^2^), a sustained ≥40% decrease in eGFR from baseline or kidney death was used as the primary or secondary prespecified kidney composite outcome, respectively [9, 10]. Time to a sustained ≥57% decrease in eGFR (equivalent to doubling of serum creatinine), time to kidney failure, or kidney death was a prespecified secondary outcome in both trials [8]. A sustained ≥40% decrease or ≥57% decrease in eGFR was defined as from baseline over ≥4 weeks [8].

### Description of the model

A full description of the development and external validation of the Klinrisk model has been reported previously, and the model was developed and validated in compliance with the Transparent Reporting of a multivariable prediction model for Individual Prognosis Or Diagnosis (TRIPOD) checklist [7]. The machine learning model was the random forest model using the R package Fast Unified Random Forest for Survival, Regression and Classification using a survival forest with right-censored data (randomForestSRC). This model was developed using variables identified in previous datasets of patients with CKD or at risk of CKD and subsequently trained using laboratory-linked administrative data from Manitoba, Canada, between 1 April 2006 and 31 December 2018, and externally validated using the Alberta Health database from Alberta, Canada, for the outcomes of kidney failure (defined as <10 ml/min/1.73 m^2^) or a ≥40% decrease in eGFR. Time horizons of 2, 3 and 4 years were selected to stay close to the overall median follow-up date within the FIDELITY dataset (3.0 years) [8].

### Variables

Variables included in the model were age (years), sex (male or female), eGFR (ml/min/1.73 m^2^), UACR (mg/mmol) and the results of 18 other laboratory analyses from chemistry panels, including creatinine, urea, sodium, potassium, bicarbonate and others such as bilirubin, and values from complete blood cell count panels including haemoglobin and platelet count.

The primary composite outcome included a ≥40% decrease in eGFR, kidney failure (defined as end-stage kidney disease [initiation of long-term dialysis for ≥90 days or kidney transplant] or eGFR <15 ml/min/1.73 m^2^) or kidney death, and the secondary composite outcome included a ≥57% decrease in eGFR, kidney failure or kidney death.

### Statistical analysis

The FIDELITY baseline characteristics were summarized with descriptive statistics.

The discrimination ability of the model to predict the outcome was evaluated using the area under the receiver operating characteristic curve (AUC) and the Brier score, annually, at 1–4 years and at the median follow-up time. Thresholds were determined based on the distribution of the data and previous publications in the field [11, 12]. Higher AUC values indicate better model performance, which translates into strong discrimination ability and high concordance between observed events and model-predicted events, whereas a lower Brier score indicates improved accuracy of probabilistic predictions. Calibration plots were produced by decile comparing observed risk and model-predicted risk. Both discrimination and calibration were evaluated in the overall population and stratified by treatment assignment (finerenone versus placebo). Kidney Disease: Improving Global Outcomes (KDIGO) heatmap categories were used as the reference standard.

Risk groups were formed based on the predicted risk from Klinrisk of a ≥40% decrease in eGFR or kidney failure. Four groups were defined based on the first three quantiles (Q1: minimum–25th percentile; Q2: 25th percentile–median or 50th quantile; Q3: median–75th percentile; and Q4: 75th percentile–maximum). Incidence rates were estimated for the risk categories and expressed per 100 patient-years. The interaction between the predicted risk and treatment assignment effects on the outcome was evaluated with Cox proportional hazards regression analysis.

## RESULTS

### Cohort description

The FIDELITY population comprised a total of 13 026 participants with a median follow-up of 3.0 years [8]. Of these, 69.8% were male, 100% had CKD and T2D, 96.5% had hypertension, 30.7% had a history of coronary artery disease and 15.5% had a prior myocardial infarction. The mean age was 64.75 ± 9.53 years, mean eGFR was 57.6 ± 21.7 ml/min/1.73 m^2^ and median UACR was 58.2 mg/mmol (Q1: 22.4 mg/mmol; Q3: 129.6 mg/mmol) (Table 1). The population distribution according to KDIGO risk categories is shown in Supplementary Fig. 1.

**Table 1:** Baseline demographics and laboratory values of the FIDELITY population by treatment

Characteristics	Finerenone (*n* = 6519)	Placebo (*n* = 6507)	Total (*N* = 13 026)
Age (years), mean ± SD	64.70 ± 9.38	64.80 ± 9.67	64.75 ± 9.53
Sex, *n* (%)			
Male	4481 (68.7)	4607 (70.8)	9088 (69.8)
Female	2037 (31.2)	1899 (29.2)	3936 (30.2)
Blood eGFR^a^ (ml/min/1.73 m^2^), mean ± SD	57.51 ± 21.59	57.65 ± 21.75	57.58 ± 21.67
UACR (mg/mmol), median (Q1–Q3)	58.11 (22.32–127.62)	58.19 (22.40–131.47)	58.16 (22.35–129.62)
Serum or plasma albumin (g/l), mean ± SD	41.88 ± 3.32	41.81 ± 3.35	41.84 ± 3.34
Serum potassium (mmol/l), mean ± SD	4.35 ± 0.44	4.35 ± 0.44	4.35 ± 0.44
Blood haemoglobin (g/l), mean ± SD	133.45 ± 17.11	133.40 ± 17.04	133.42 ± 17.07
Serum urea nitrogen (mmol/l), mean ± SD	8.95 ± 3.77	8.94 ± 3.68	8.95 ± 3.73
Serum ALP (U/l), mean ± SD	79.83 ± 29.25	80.12 ± 29.05	79.97 ± 29.15
GGT^b^ (U/l), mean ± SD	40.30 ± 52.62	39.48 ± 52.26	39.89 ± 52.44
CKD^c^, *n* (%)	6519 (100)	6507 (100)	13 026 (100)
T2D^c^, *n* (%)	6518 (>99.9)	6506 (>99.9)	13 024 (>99.9)
Hypertension^d^, *n* (%)	6281 (96.3)	6285 (96.6)	12 566 (96.5)
Coronary artery disease^c^, *n* (%)	1990 (30.5)	2007 (30.8)	3997 (30.7)
Myocardial infarction^c^, *n* (%)	1018 (15.6)	1004 (15.4)	2022 (15.5)

a Blood eGFR measured using the CKD-EPI equation.

b Serum or plasma.

c Preferred term in MedDRA version 23.1.

d MedDRA Labelling Groupings term in MedDRA version 23.1.

ALP: alkaline phosphatase; CKD-EPI: Chronic Kidney Disease Epidemiology Collaboration; GGT: gamma-glutamyl transferase; MedDRA: Medical Dictionary for Regulatory Activities; Q: quantile; SD: standard deviation.

### Model performance and external validation

**Figure 1:**
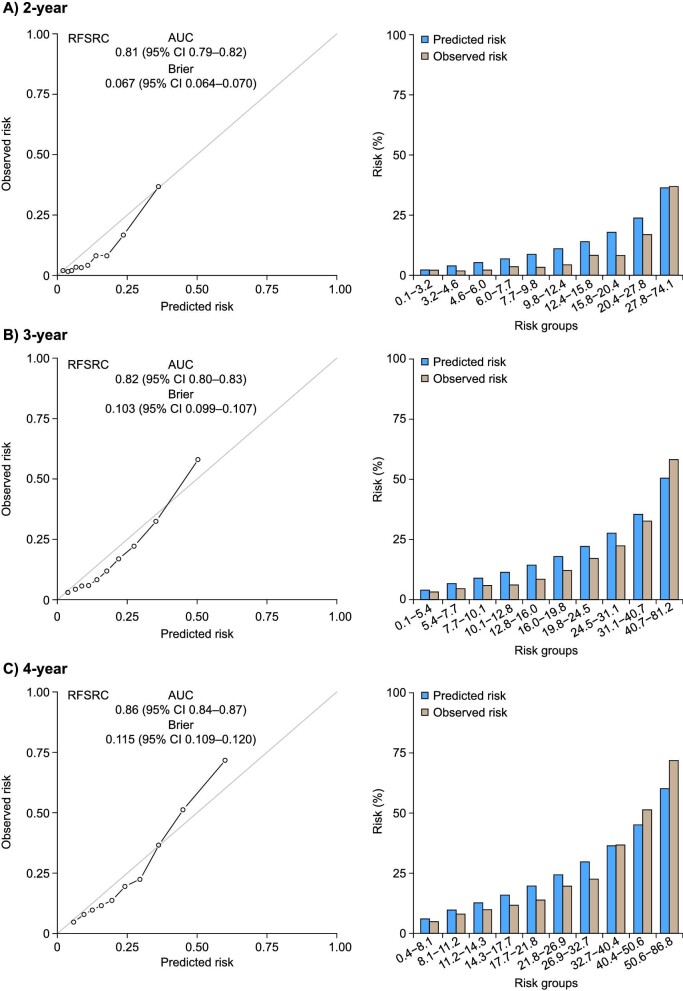
Calibration plots for the Klinrisk prediction model for the primary composite outcome (eGFR decrease ≥40% or kidney failure) at **(A)** 2, **(B)** 3 and **(C)** 4 years after randomization in the FIDELITY population. RFSRC: Random Forests for Survival, Regression, and Classification.

At 2 and 4 years, 993 and 1795 patients experienced a primary outcome event (≥40% decrease in eGFR or kidney failure), respectively. The model demonstrated good performance and predicted the primary outcome accurately in the FIDELITY population with an AUC of 0.81 [95% confidence interval (CI) 0.79–0.82] for 2-year prediction, 0.82 (95% CI 0.80–0.83) for 3-year prediction and 0.86 (95% CI 0.84–0.87) for 4‑year prediction (Table 2, Fig. 1). The Klinrisk model outperformed the KDIGO heatmap categories, where the KDIGO heatmap categories showed an AUC of 0.59 (95% CI 0.58–0.60) for 2-year prediction, 0.60 (95% CI 0.59–0.61) for 3-year prediction and 0.66 (95% CI 0.65–0.68) for 4-year prediction (Table 2, Fig. 1). Similarly, kidney failure risk equation (KFRE) model categories showed an AUC of 0.76 (95% CI 0.75–0.78) for 2-year prediction, 0.77 (95% CI 0.76–0.79) for 3-year prediction and 0.83 (95% CI 0.82–0.85) for 4-year prediction (Table 2).

**Table 2:** AUC scores for years 2–4 for KDIGO heatmap categories, KFRE and Klinrisk models

Time frame (years)	KDIGO heat map, AUC (95% CI)	KFRE model, AUC (95% CI)	Klinrisk model, AUC (95% CI)
2	0.59 (0.58–0.60)	0.76 (0.75–0.78)	0.81 (0.79–0.82)
3	0.60 (0.59–0.61)	0.77 (0.76–0.79)	0.82 (0.80–0.83)
4	0.66 (0.65–0.68)	0.83 (0.82–0.85)	0.86 (0.84–0.87)

Calibration was appropriate at 2, 3 and 4 years in the FIDELITY population, with a Brier score for 2-year prediction of 0.067 (95% CI 0.064–0.070), for 3-year prediction of 0.103 (95% CI 0.099–0.107) and for 4-year prediction of 0.115 (95% CI 0.109–0.120). Similar discrimination accuracy was seen for the secondary composite outcome (≥57% decrease in eGFR or kidney failure) at 3 years [AUC 0.88 (95% CI 0.87–0.90)].

**Figure 2:**
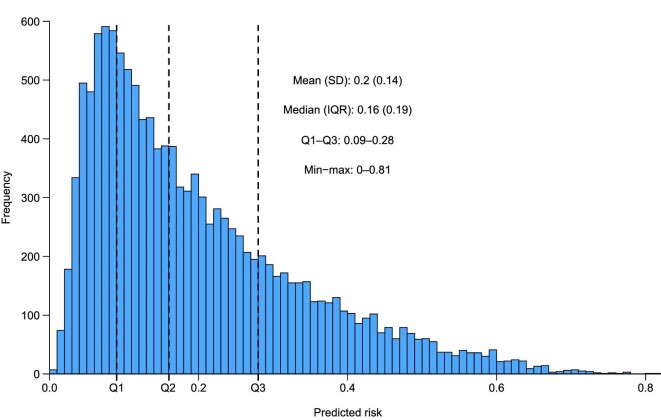
Histogram of predicted risk by the Klinrisk prediction model for the primary composite outcome (eGFR decrease ≥40% or kidney failure) in the FIDELITY population over 3 years. Patients were divided into 10 groups using the deciles of the predicted risk from the model. Thresholds were determined based on the distribution of the data and previous publications in the field [11, 12]. IQR: interquartile range; min: minimum; max: maximum; Q: quantile; SD: standard deviation.

To assess the risk categorization of the model within the FIDELITY dataset, incidence rates for the primary composite outcome were examined by risk group, defined by the range of predicted risk from Q1 (minimum–25th percentile) to Q4 (75th percentile–maximum). Risk groups were defined by the range of the predicted risk as shown in Fig. 2 and Table 3. Incidence rates for the primary composite outcome corresponded with the assigned risk group (i.e. lower for low-risk groups and higher for high-risk groups). Incidence rates were lower in the finerenone group versus placebo across all risk groups. No evidence of interaction between treatment and risk was identified (*P* = .31) (Supplementary Table 1). In addition, no major deviation was observed in calibration across age groups (Supplementary Fig. 2).

**Table 3:** Incidence rates by risk group for primary composite endpoint (decrease in eGFR of ≥40% or kidney failure)

Group	Patients (*n*)	Events (*n*)	Incidence rate per 100 PY	95% CI
Finerenone				
Q1	1657	199	4.40	3.83–5.05
Q2	1663	185	4.13	3.58–4.77
Q3	1600	220	5.15	4.52–5.88
Q4	1599	228	5.59	4.91–6.36
Placebo				
Q1	1600	228	5.16	4.53–5.87
Q2	1593	214	5.03	4.40–5.75
Q3	1656	235	5.40	4.76–6.14
Q4	1658	286	6.76	6.02–7.59

Risk groups are defined by the range of predicted risk. Q1: first quantile (minimum–25th percentile); Q2: second quantile (25th percentile–median); Q3: third quantile (median–75th percentile); Q4: fourth quantile (75th percentile–maximum). PY: patient-years; Q: quantile.

### Subgroup analysis

Although slightly lower compared with placebo, the discrimination of the model when assessing those in the finerenone group across the same time period was good, with AUCs from the receiver operating characteristics curves of 0.77 (95% CI 0.75–0.80) at 2 years, 0.80 (95% CI 0.78–0.82) at 3 years and 0.84 (95% CI 0.82–0.86) at 4 years for finerenone, and 0.84 (95% CI 0.82–0.86) at 2 years, 0.83 (95% CI 0.82–0.85) at 3 years, and 0.87 (95% CI 0.85–0.89) at 4 years for placebo.

## DISCUSSION

In this external validation of the Klinrisk model, we demonstrate that the model originally developed in the general population, accurately predicted CKD progression events in patients with CKD and T2D. This analysis provides a comprehensive validation of the Klinrisk model in a well-characterized, global clinical trial population across all stages of CKD with centrally adjudicated clinical events. These findings add to the external validity of the model and highlight the need for studies on its clinical implementation.

The Klinrisk model was developed using a cohort of community-dwelling adults with a mean age of 59 years, mean eGFR of 82 ml/min/1.73 m^2^ and median UACR of 1.1 mg/mmol. Although the majority of this population had early-stage CKD, a significant proportion were disease-free at baseline, and the overall event rate was only 7.8% at 5 years [7]. In contrast, the FIDELITY dataset represented a population at high risk of CKD progression, with a mean age of 65 years, mean eGFR of 58 ml/min/1.73 m^2^ and median UACR of 58 mg/mmol [8]. Despite these differences, the Klinrisk model appears to both discriminate and calibrate well in the FIDELITY population.

Predictive models for CKD progression have been tested in similar populations. In the Action to Control CardiOvascular Risk in Diabetes (ACCORD) trial, a time-varying Cox model demonstrated good discrimination [AUC 0.745 (95% CI 0.723–0.763)] and calibration [Brier score 0.0923 (95% CI 0.0873–0.0965)] performance in patients with CKD and T2D [13]. More recently, a proprietary machine model (KidneyIntelX) was tested in a subpopulation of the CANagliflozin cardioVascular Assessment Study (CANVAS) trial, and KidneyIntelX successfully risk stratified a large multinational external cohort for risk of CKD progression [14]. A new regression-based model from the CKD Prognosis Consortium, for patients with or without diabetes with recently developed CKD (eGFR ≥60 ml/min/1.73 m^2^), also included data from randomized trials and observational studies, finding good discrimination in a pooled validation [15].

Our current work on the Klinrisk model builds on previous and ongoing work on the KFRE, which uses the same principles of applying routinely collected laboratory data to predict CKD progression [16]. However, there are several notable differences. First, the KFRE is not accurate in early-stage CKD [17]. High-risk patients with early-stage disease (e.g. a 50-year-old male with an eGFR of 80 ml/min/1.73 m^2^ and UACR of 100 mg/mmol) would have a low kidney failure risk using the KFRE (0.2% at 5 years) but a very high risk of progression defined by a ≥40% decrease in eGFR (25.8% by the Klinrisk model). As such, identification of these patients as high risk early in their disease course and appropriate treatment with renin–angiotensin–aldosterone system inhibitors, sodium–glucose co-transporter-2 inhibitors and finerenone could prevent a lifetime risk of kidney failure.

Recent work by investigators from the United Kingdom suggested that automatic identification of individuals at high risk of CKD progression and CV risk is important for improving CKD care [18]. The investigators performed a systematic review and realist synthesis to develop an integrated model of intervention mechanisms to improve the delivery of CKD care. Their model suggested that automatic detection of high-risk cases in primary care, alongside education, when integrated into existing workflows can improve kidney and CV outcomes. The Klinrisk model, with its use of routinely collected laboratory data and ability to integrate with both laboratory data or electronic medical records, can fill this implementation gap and improve outcomes in primary care where most CKD cases reside. In most countries, interventions targeted at primary care are the only feasible path to improving CKD-related population health because nephrology resources are limited.

We compared the performance of the Klinrisk model with the KDIGO heatmap and the KFRE instead of the KidneyIntelX model (as we did not have access to the biomarkers required for KidneyIntelX) and found evidence of substantial improvement in discrimination. The KDIGO heatmap can be seen as a standard reference tool in clinical practice; however, the Klinrisk model outperforms the KDIGO heatmap in prediction accuracy. This is because the heatmap provides population-based risk categories [4], which are necessary, but insufficient, for individual risk stratification. Heterogeneity in the predicted risk of adverse outcomes has been found within each category, with overlap between categories [19]. In each heatmap risk category, there can be large variations in the factors associated with the risk of kidney failure and CKD progression [20]. Models that provide absolute risk at the individual level can go beyond the heatmap and identify patients at the greatest risk.

There was no evidence of interaction between treatment and risk for the primary outcome in the current analysis (*P* = .31). This suggests all patients included in FIDELITY, regardless of CKD stage, had a similar benefit, on a relative scale, from the intervention. This is consistent with data from sodium–glucose co-transporter-2 inhibitor trials in diabetes and heart failure [21]. However, the benefit would be larger on the absolute scale than the relative scale in individuals at higher risk.

There are several implications of our findings. From a clinical perspective, the Klinrisk model can be used to identify patients with early-stage CKD and T2D but a high risk of progression, who can be treated accordingly to delay or prevent progression to kidney failure. Currently, most of these patients are unaware of their disease and are underdiagnosed. From a research perspective, the model may be useful for identifying intermediate- or high-risk patients for inclusion in clinical trials to enhance event rates with 2–3 years of follow-up. From a health services perspective, payers can use the model to identify patients with the highest CKD-specific costs, and who may benefit from early treatment. Although we did not evaluate the association between risk and cost of care, findings from the KFRE strongly support an association between CKD progression risk score and cost of care [22].

This analysis has several limitations. The FIDELITY population consists of patients from two randomized clinical trials and is therefore highly selected. As a result, further analyses using other large observational datasets should be considered. In addition, our model only predicts the progression of CKD (assessed by the primary composite outcome of a ≥40% decrease in eGFR, kidney failure or kidney death), whereas many patients with CKD and T2D are at higher risk of CV events than kidney failure [23–25]. This is important because finerenone has clear CV benefits that may or may not be distributed along the same risk categories as CKD progression benefits [8]. Therefore, refitting of the model to predict CV events or validation of a de novo CV model is needed to predict cardiorenal risk and potential cardiorenal benefit from therapy in this population. Furthermore, the severity of outcomes in the primary composite outcome differed (e.g. eGFR decrease versus kidney failure); however, it was not possible to explore these separately due to the study designs of the FIDELIO-DKD and FIGARO-DKD trials. Despite this, kidney composite outcomes were primarily driven by a ≥40% decrease in eGFR because patients with an eGFR of <25 ml/min/1.73 m^2^ were excluded from FIDELITY. Given that FIDELITY includes a large population of patients with CKD and T2D across the spectrum of CKD severity, using this dataset to develop a prognostic model to estimate CKD progression and response to treatment should be considered in the future. Moreover, the Klinrisk model requires commonly ordered laboratory data, which although accessible prospectively, may not be available retrospectively. However, the initial validation studies confirmed the model can be highly accurate with missing data by using imputation algorithms with a preserved ability to score as long as >7 of 20 laboratory variables are present. Although the model is externally validated, additional studies are needed to address the impact of the model predictions and associated clinical decision support on quality and processes of CKD care and downstream outcomes. In addition, the Klinrisk model contains 20 routinely collected laboratory variables. Future iterations of the model could be simplified to require fewer variables without sacrificing model performance. Finally, cost-effectiveness analyses that evaluate the benefit of a risk-based treatment strategy to current standard of care will be needed prior to widespread adoption of prediction tools.

In conclusion, our findings demonstrate the accuracy and potential utility of a machine learning risk-prediction model, Klinrisk, based on routinely collected laboratory data for identifying patients at the highest risk of CKD progression early in their course of disease. Prospective studies implementing these models in electronic health records and laboratory information systems to identify and treat patients with high-risk CKD and T2D are needed.

## Supplementary Material

sfae052_Supplemental_Files

## Data Availability

Availability of the data underlying this publication will be determined according to Bayer's commitment to the EFPIA/PhRMA ‘Principles for responsible clinical trial data sharing’. This pertains to scope, time point, and process of data access. As such, Bayer commits to sharing upon request from qualified scientific and medical researchers’ patient-level clinical trial data, study-level clinical trial data, and protocols from clinical trials in patients for medicines and indications approved in the United States (US) and European Union (EU) as necessary for conducting legitimate research. This applies to data on new medicines and indications that have been approved by the EU and US regulatory agencies on or after 01 January 2014. Interested researchers can use www.vivli.org to request access to anonymized patient-level data and supporting documents from clinical studies to conduct further research that can help advance medical science or improve patient care. Information on the Bayer criteria for listing studies and other relevant information is provided in the member section of the portal. Data access will be granted to anonymized patient-level data, protocols, and clinical study reports after approval by an independent scientific review panel. Bayer is not involved in the decisions made by the independent review panel. Bayer will take all necessary measures to ensure that patient privacy is safeguarded.
