# Supplementary material for: Validation of the Klinrisk chronic kidney disease progression model in the FIDELITY population
Source: Clin Kidney J. 2024 Mar 6;17(4):sfae052. doi: 10.1093/ckj/sfae052 (PMC11033844; doi:10.1093/ckj/sfae052)
Supplement: sfae052_Supplemental_Files [file sfae052_supplemental_files.zip › 240103_FIDELITY Klinrisk MS Suppl Figure 1.pdf]

GFR categories (ml/min/1.73 m²)	Data presented as n (%)	Albuminuria categories (mg albumin/g creatinine)		
		**A1** Normal to mildly increased	**A2** Moderately increased	**A3** Severely increased
		0–<30	30–<300	≥300–≤5000
**G1**	≥90	**13 (<0.1)**	**198 (1.5)**	**1108 (8.5)**
**G2**	60–89	**51 (0.4)**	**1043 (8.0)**	**2780 (21)**
**G3a**	45–59	82 (0.6)	1389 (11)	1962 (15)
**G3b**	30–44	68 (0.5)	1230 (9.4)	2206 (17)
**G4**	15–29	16 (0.1)	239 (1.8)	635 (4.9)
