# Supplementary material for: Validation of the Klinrisk chronic kidney disease progression model in the FIDELITY population
Source: Clin Kidney J. 2024 Mar 6;17(4):sfae052. doi: 10.1093/ckj/sfae052 (PMC11033844; doi:10.1093/ckj/sfae052)
Supplement: sfae052_Supplemental_Files [file sfae052_supplemental_files.zip › 240221_Supplementary materials_Draft 4.docx]

**Supplementary Table 1.** Cox regression models with treatment by risk interaction

| **Outcome** | **Parameter** | **Parameter estimate** | **Standard error** | **P-value** |
| --- | --- | --- | --- | --- |
| Onset of kidney failure, a sustained decrease of eGFR ≥40% from baseline over at least 4 weeks, or renal death | Treatment | –0.09 | 0.08 | 0.28 |
|  | Risk | 1.10 | 0.21 | <0.0001 |
|  | Treatment*risk | –0.33 | 0.32 | 0.31 |
| Onset of kidney failure, a sustained decrease of eGFR ≥57% from baseline over at least 4 weeks, or renal death | Treatment | –0.33 | 0.12 | 0.01 |
|  | Risk | 1.43 | 0.31 | <0.0001 |
|  | Treatment*risk | 0.28 | 0.46 | 0.54 |

eGFR, estimated glomerular filtration rate.

**Supplementary Figure 1.** Baseline GFR and UACR (KDIGO categories) in the FIDELITY population^a^


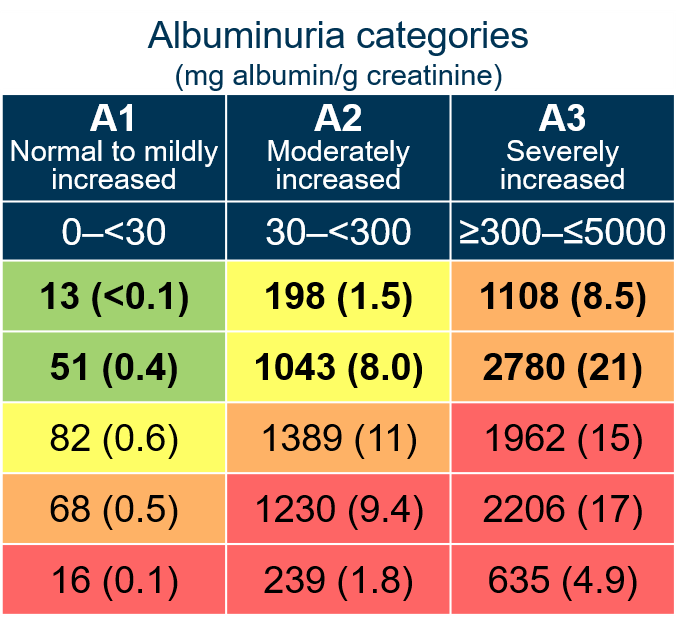

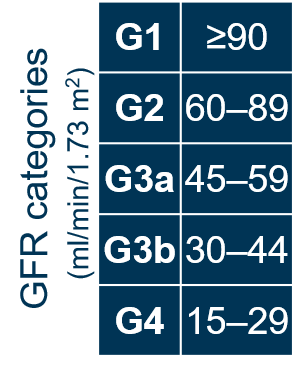


Data presented as n (%)

^a^Data were missing for 3 patients.

GFR, glomerular filtration rate; KDIGO, Kidney Disease Improving Global Outcomes; UACR, urine albumin-to-creatinine ratio.

**Supplementary Figure 2.** AUC, Brier score, and calibration plots of age distribution in **(A)** <59 years age group, **(B)** 59–71 years age group, and **(C)** >71 years age group in the FIDELITY population


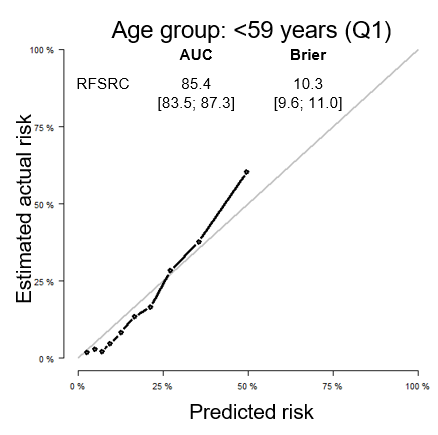


**A)**

**B)**


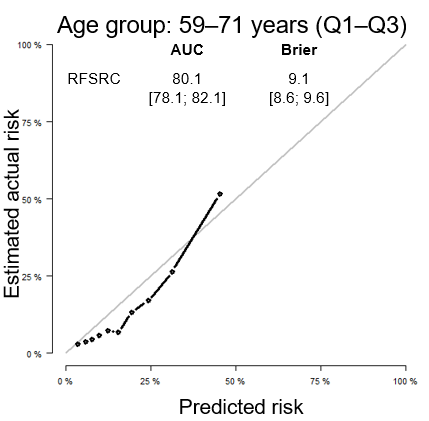


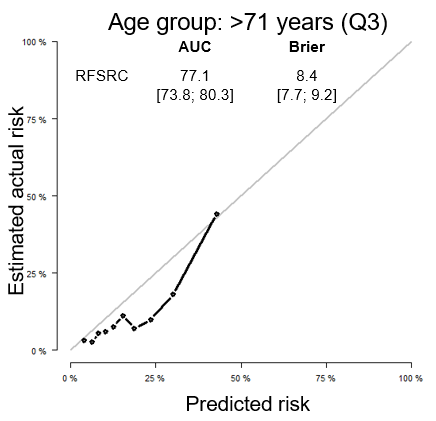


**C)**

AUC, area under the receiver operating characteristic curve; Q, quartile; RFSRC, Random Forests for Survival, Regression, and Classification.
