# Supplementary figures and images for: Validation of the Klinrisk chronic kidney disease progression model in the FIDELITY population
Source: Clin Kidney J. 2024 Mar 6;17(4):sfae052. doi: 10.1093/ckj/sfae052 (PMC11033844; doi:10.1093/ckj/sfae052)

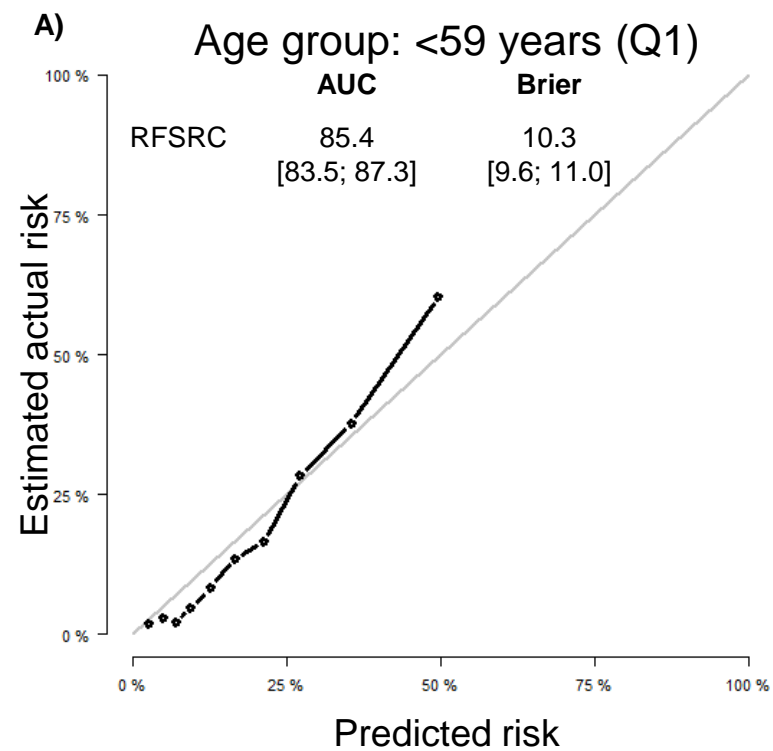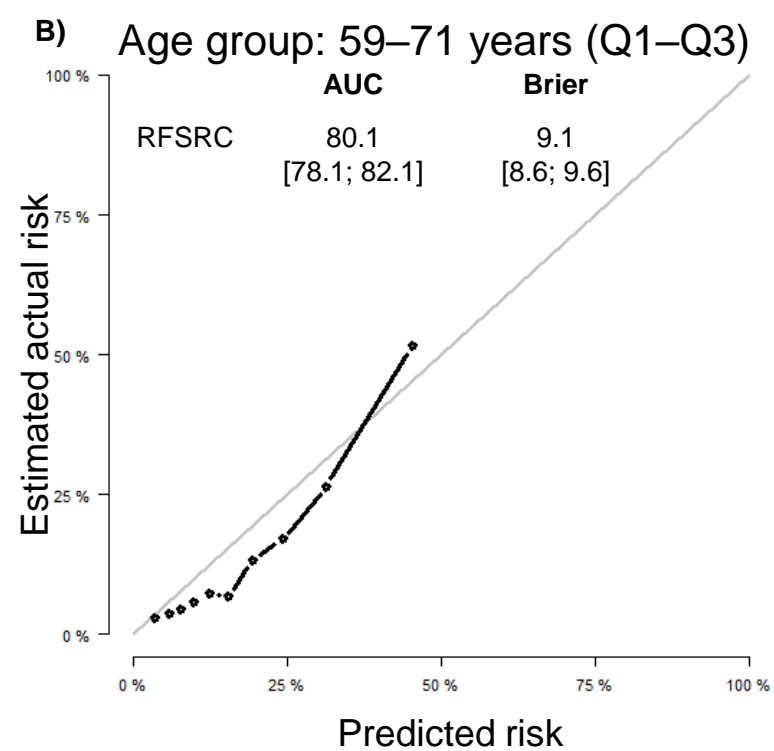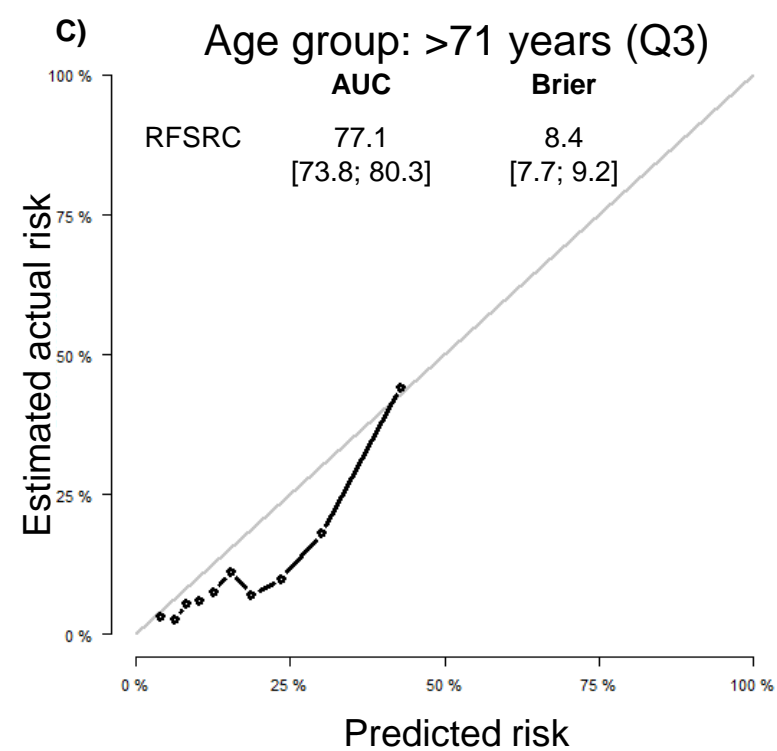

Supplement: sfae052_Supplemental_Files [file sfae052_supplemental_files.zip › 240103_FIDELITY Klinrisk MS Suppl Figure 2.pdf]
